# The Impact of Digitalization and Information and Communication Technology on the Nature and Organization of Work and the Emerging Challenges for Occupational Safety and Health

**DOI:** 10.3390/ijerph22030362

**Published:** 2025-02-28

**Authors:** Izuchukwu Chukwuma Obasi, Chizubem Benson

**Affiliations:** CERIDES—Center of Excellence in Risk and Decision Sciences, European University Cyprus, Nicosia 2404, Cyprus; cb161587@students.euc.ac.cy

**Keywords:** ICT, Industry 4.0, regulatory frameworks, emerging risks, workplace safety

## Abstract

Digitalization, driven by the widespread adoption of information and communication technology (ICT), reshapes occupational safety (OSH). This study examines emerging OSH risks linked to digitalization, assessing its benefits and challenges. Through a comprehensive literature review, key technologies influencing OSH are identified, their effects are categorized, and mitigation strategies are proposed. While ICT enhances workplace safety through improved monitoring and decision-making, it also introduces risks such as stress and information overload. The findings emphasize the need for further research on long-term impacts and effective risk management. This paper contributes to the field by highlighting ICT’s positive and negative implications on OSH and underscoring the importance of responsible technology adoption. The insights presented are valuable for policymakers, researchers, and industry practitioners committed to fostering a safe and healthy work environment.

## 1. Introduction

The rapid advancement of digitalization has significantly transformed various industries, marking the onset of the Fourth Industrial Revolution (Industry 4.0) [1]. This paradigm shift encompasses a convergence of technologies, including artificial intelligence (AI), the Internet of Things (IoT), big data, cyber–physical systems (CPSs), and cloud computing, all of which are reshaping modern workplaces and organizational structures [2]. Unlike previous industrial revolutions, which focused primarily on mechanization, electrification, and digital computing, Industry 4.0 integrates these technologies into interconnected systems that enhance efficiency, productivity, and decision-making [3,4]. However, while digitalization offers substantial benefits, it also introduces complex challenges concerning occupational health and safety (OSH).

Adopting digital technologies in the workplace has significantly transformed job roles, working conditions, and occupational health risk factors. Smart technologies enhance safety through real-time monitoring, predictive maintenance, and automation [5,6,7], and also introduce new challenges. These include increased work intensification, ergonomic issues from prolonged screen use, cybersecurity threats, and psychosocial stressors associated with constant connectivity [8]. However, much of the existing research has focused on the economic and productivity gains of Industry 4.0, with relatively little attention given to its impact on worker well-being and safety [9].

A critical gap in the literature concerns the nuanced effects of digitalization on different workforce segments. Much of the existing research centers on high-skilled professionals, overlooking how digital transformation affects workers across various industries and job classifications. Moreover, organizational factors, such as workplace policies, job autonomy, and regulatory frameworks, significantly mediate the impact of digitalization on health and safety. For instance, the widespread adoption of ICT has led to “omnipresent connectivity” [10], enabling work beyond traditional office settings. However, this shift has also given rise to the “autonomy paradox”, where workers experience diminished control over their schedules due to expectations of constant availability [11,12]. These evolving work dynamics necessitate a more comprehensive examination of digital work intensification and its implications for OSH.

Furthermore, while regulatory frameworks exist to safeguard worker health, they often struggle to keep pace with rapid technological advancements. The effectiveness of current OSH policies in addressing emerging risks associated with Industry 4.0 remains an open question. For instance, legislative initiatives, such as the European Union’s “Right to Disconnect” policy, aim to mitigate the adverse effects of excessive ICT use, yet their adoption and enforcement vary across industries and regions [13]. As digitalization accelerates, there is an urgent need to assess whether existing OSH regulations are adequate or require significant adaptation to safeguard worker well-being.

Given these considerations, this study aims to critically evaluate the implications of digitalization and ICT adoption on occupational health and safety. By conducting a systematic literature review, this research seeks to (1) identify both the benefits and emerging risks associated with digital technologies in the workplace, (2) assess the effectiveness of current OSH regulatory frameworks, and (3) highlight key research gaps and future directions. The following key questions will guide the review: Have we thoroughly evaluated ICT and digitalization’s positive and negative OSH consequences? Is there a risk of losing the progress made through proactive measures? Are there valid reasons for concern? This foresight project addresses these questions by providing reliable and high-quality information about emerging OSH risks resulting from digitalization, changes in ICT, and their impact on work. The following sections will outline the methodology employed, present an analysis of the relevant literature, and discuss the broader implications of digital transformation on worker safety. Additionally, policy recommendations will be proposed to inform future research and regulatory developments in this rapidly evolving domain.

## 2. Methodology

This research examined the impacts of adopting ICT and digitalization on occupational safety and health (OSH), considering both the positive and negative influences. The study aimed to identify potential new and emerging OSH risks associated with these technological advancements and highlight future research opportunities.

The systematic literature review was the most suitable methodology to address the research questions. This approach provides a comprehensive understanding of the research topic by ensuring replication, transparency, and minimizing bias, thus enhancing the reliability of the research [14]. By employing this method, we can identify gaps in the research and opportunities for future investigations [15].

This review adheres to the Preferred Reporting Items for Systematic Reviews and Meta-Analyses (PRISMA) framework, with slight modifications to align with the study’s specific objectives [16]. Insights from previous systematic reviews inform the methodology and keyword selection process [8,17,18].

**Figure 1:**
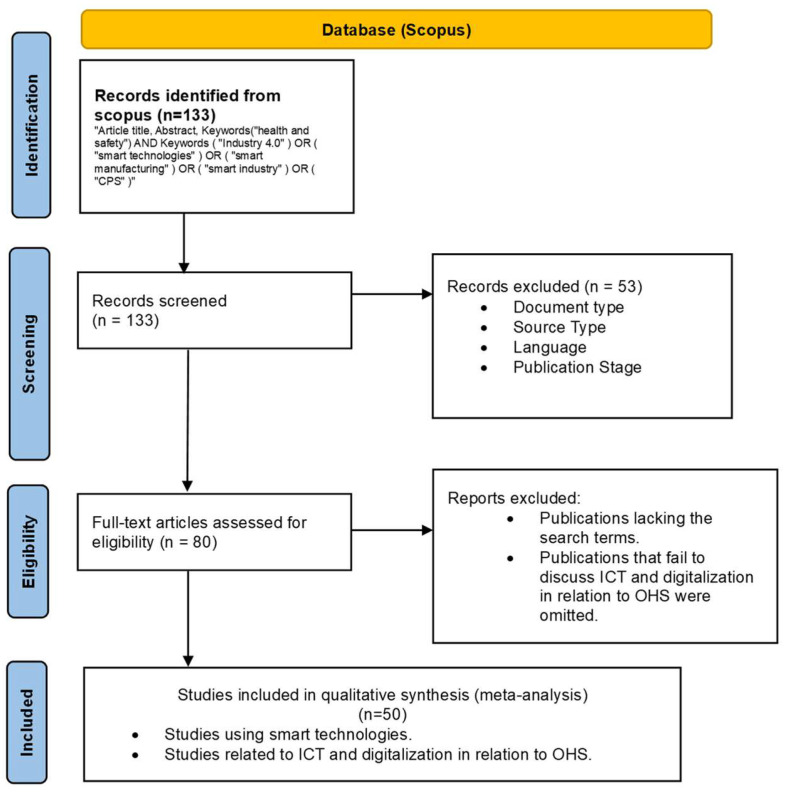
Methodological flowchart.

Figure 1 illustrates the research methodology, structured according to the PRISMA flow diagram, detailing the number of documents retained at each selection stage. The primary objective of this review is to examine ICT and digitalization’s positive and negative OSH consequences. This study aims to categorize the broader research domain and identify specific research opportunities within each category. The collection process strictly adheres to PRISMA guidelines to ensure the inclusion of high-quality articles.

This study proposes a robust search strategy to ensure comprehensive coverage while minimizing false positives, thereby maintaining the accuracy of bibliometric findings. Relying solely on a term-based search strategy may not effectively achieve the research objectives. For example, using “health and safety” as the only search term in titles, abstracts, and keywords within articles indexed by Scopus Core Collection may overlook significant literature while generating irrelevant results. This approach lacks the specificity and sensitivity needed to capture the relevant literature effectively.

### 2.1. Search Strategy

Drawing from the existing literature, our search strategy examined the potential impacts of ICT and digitalization on occupational safety and health (OSH). Given the significance of these technological advancements, we identified the need for a comprehensive literature review to evaluate their benefits and risks in the OSH domain.

The search string was carefully designed with the understanding that ICT and digitalization encompass a wide range of technologies commonly applied in OSH. To ensure relevant and comprehensive coverage, the search strategy incorporated the following query:

“Article title, Abstract, Keywords (’health and safety’) AND Keywords (’Industry 4.0′) OR (’smart technologies’) OR (’smart manufacturing’) OR (’smart industry’) OR (’CPS’).”

### 2.2. Risk of Bias Assessment

Ensuring the credibility of a literature review requires a systematic approach to minimize bias in article selection and analysis. This study followed best practices for systematic reviews, addressing selection, analysis, and publication bias.

A predefined review protocol with clear inclusion and exclusion criteria was used to reduce selection bias, adhering to the PRISMA framework for transparent screening and reporting. Backward and forward citation tracking helped identify overlooked studies.

A structured data extraction framework with standardized coding ensured objective analysis, minimizing researcher subjectivity. Contradictory findings were included to prevent confirmation bias and enhance reliability.

Database selection bias was mitigated using Scopus while cross-checking key references against Google Scholar and ScienceDirect. Despite these efforts, language bias remained a limitation, as only English language sources were included. High-impact studies’ translated abstracts were reviewed, but future research should incorporate multilingual databases.

An independent researcher reviewed the selection process to enhance objectivity, and diverse viewpoints were included to provide a balanced discussion on digitalization’s impact on OSH.

### 2.3. Selected Studies

As noted by Chadegani et al. [19], the Scopus database was chosen for its comprehensive coverage of journals and publications. The search string was then applied to Scopus, identifying 133 articles. The screening process was designed to assess the effectiveness of the search string in retrieving enough studies to adequately address the research questions.

We categorized these papers based on the criteria outlined in Table 1.

The refined search strategy is illustrated in Figure 1. Subsequently, the chosen articles underwent a rigorous screening process, which involved a meticulous review of their titles and abstracts. Importantly, inclusion in this review was contingent upon meeting specific criteria, which included the following stipulations: The study’s emphasis should center on publications addressing ICT and digitalization in connection with health and safety. Additionally, only final peer-reviewed journal articles published in English were eligible for inclusion, while review articles, conference proceedings, book series, books, and reports were excluded from consideration.

Following the criteria, a comprehensive review of the complete texts excluded 83 articles. Consequently, 50 pertinent papers spanning publication years from 2014 to 2024 were identified for further analysis.

Building on the initial findings from the literature search, the discussion was expanded to explore the broader implications of ICT and digitalization on worker health and safety. This analysis encompassed various technological domains outlined in Table 2, including artificial intelligence, big data, cyber–physical systems, computer networks, cobotics, the Internet of Things, and computer simulations.

Additionally, the discussion addressed four critical aspects of occupational health and safety (OSH): (1) work organization, (2) OSH legislative and regulatory frameworks, (3) OSH management systems, and (4) occupational risk management [8]. A broader selection of the literature published since 2014 was incorporated to provide a more comprehensive perspective and strengthen the analysis, offering deeper insights and additional supporting evidence.

In conclusion, a wide range of recommendations concerning implementing occupational health and safety (OSH) measures within information and communication technology (ICT) and workplace digitalization were generated and thoroughly examined.

## 3. Result

Information and communication technology (ICT) and digitalization have significantly transformed the nature and structure of work. This review article aims to systematically compile and organize the existing research on the effects of digital transformation on work dynamics and organization. The goal is to offer valuable and critical insights into this evolving field. Section 3.1 presents an overview of the quantitative aspects of the reviewed literature and Section 3.2 identifies the key ICTs driving digitalization.

### 3.1. Descriptive Analysis of Literature

**Figure 2:**
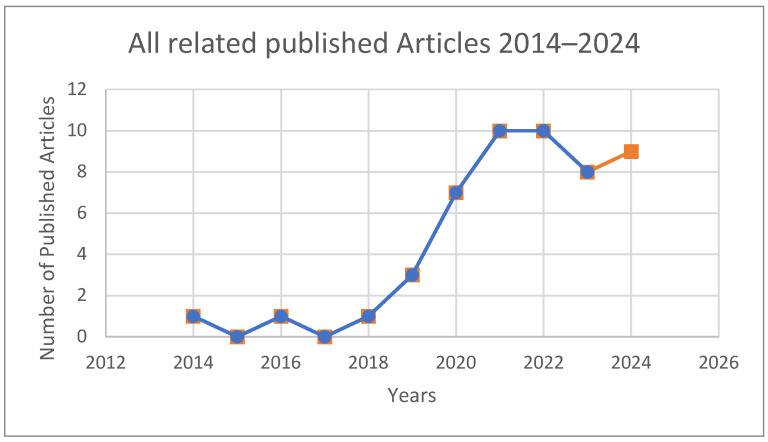
Distribution of articles by year of publication.

Figure 2 illustrates the annual publication trends showcasing a growing research interest in the intersection of ICT, digitalization, and occupational safety and health (OSH) since 2014. Between 2014 and 2018, the number of related publications remained relatively low, with minor fluctuations. However, from 2018 onward, there was a steady increase in publications, culminating in a significant surge between 2020 and 2022, where the annual count peaked. This upward trend reflects the expanding adoption of technologies such as additive manufacturing and IoT in manufacturing. The sustained interest in this domain can be attributed to technological advancements and the increasing integration of smart systems in industrial safety decision-making. This ongoing research emphasis underscores the growing relevance of these topics in recent years, with continued scholarly attention observed through 2024.

Table 3 presents the distribution of reviewed papers across various journals, highlighting those with more than two contributions to the research domain. Unlike other studies where a dominant journal emerges, the distribution here is relatively fragmented. *Sustainability Switzerland* leads with four published articles, followed by *Computers and Industrial Engineering* with three. Other journals, including the International *Journal of Advanced Manufacturing Technology*, *IEEE Access*, *American Journal of Industrial Medicine*, and *Safety Science*, each contributed two articles.

The relatively few publications per journal suggest that research on ICT, digitalization, and occupational safety and health (OSH) remains distributed across multiple disciplines rather than concentrated in a single, leading journal. This dispersion indicates the interdisciplinary nature of the field and the evolving interest in integrating smart systems into safety decision-making within Industry 4.0.

**Figure 3:**
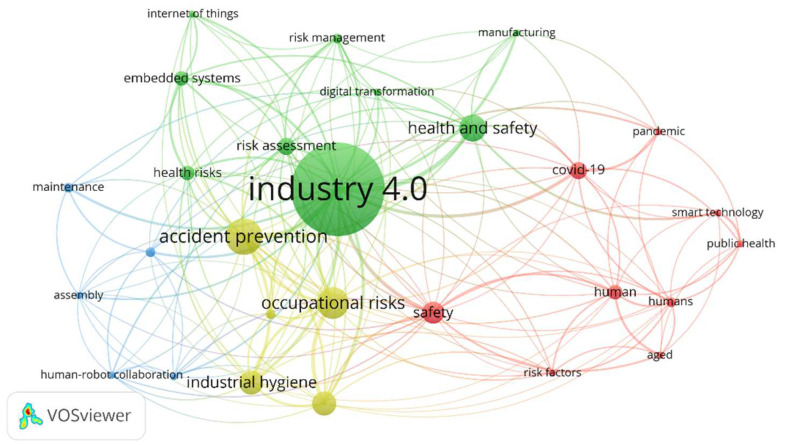
Authors’ keyword visualization.

Keywords serve as pivotal indicators of a study’s core content, explaining the thematic focus within the study domain. The keywords are analyzed using the VOSviewer set at fractional counting, and then the minimum number of occurrences of the keyword set is set at 3. Out of the 620 keywords, 28 meet the threshold. The findings from the keyword analysis are presented in Figure 3. The central keyword, “Industry 4.0”, emerges as the most dominant, indicating its significant role in the research landscape. Closely linked terms such as “health and safety”, “risk assessment”, and “accident prevention” underscore the increasing focus on occupational safety and health (OSH) in industrial settings. The green cluster primarily revolves around digital transformation, IoT, risk management, and manufacturing, suggesting the role of smart technologies in safety improvements. The yellow cluster focuses on accident prevention, artificial intelligence, and occupational risks, highlighting research on predictive safety measures. The blue cluster is centered on robotics, automation, and human–robot collaboration, reflecting advancements in workplace automation. The red cluster is heavily associated with COVID-19, safety, public health, and smart technology, revealing the pandemic’s impact on safety research in industrial environments. The interconnected nature of these clusters suggests a strong relationship among Industry 4.0 technologies, workplace safety, and automation. The presence of terms like “artificial intelligence”, “embedded systems”, and “occupational health and safety” indicates a growing interdisciplinary approach to mitigating risks in industrial settings. This analysis reinforces that integrating smart systems into industrial environments is a key trend in modern safety research.

Table 4 presents some of the papers within the literature review. The authors have categorized these documents based on the specific ICT and digitalization technologies discussed in the study. Notably, there has been a significant increase in scientific publications related to ICT and digitalization. However, it is observed that only a few of these publications address OSH concerns in a meaningful manner. Among the identified documents, the majority primarily focus on examining the correlation between ICT, digitalization, and OSH. This observation aligns with similar studies [26]. It may be attributed to the early implementation phase of certain ICT-related technologies, which makes it challenging to assess their impacts on OSH. Additionally, it is essential to acknowledge that symptoms experienced by workers may take some time to manifest, even though many of these technologies have already been adopted by various companies for several decades.

### 3.2. ICT and Digitalization Technologies

Since the late 20th century, there has been a remarkable surge in the development of electronics and information and communication technologies (ICTs). This progress has focused on miniaturizing electronic components and devices, allowing computing systems to become more portable and supporting the creation of wireless networks that connect physical objects and devices [50]. When interconnected, these devices can function seamlessly as a cohesive system. At the same time, recent breakthroughs in sensor technologies have made it easier, more reliable, and cost-effective to measure a wide range of physical, chemical, and spatial properties of real-world objects and environments.

These technological advancements have given rise to new and interconnected concepts, which are often discussed in the literature using terms such as Artificial Intelligence (AI) [35], cyber–physical systems (CPSs) [45], Virtual Reality (VR) [49], cobotics (CO) [44], Augmented Reality (AR) [43], big data (BD) [8], Internet of Things (IoT) [39], and simulation (SM) [8]. These concepts overlap to some extent and are intertwined, representing significant developments in the field.

Table 5 comprehensively analyzes key ICTs, outlining their advantages and disadvantages in various industrial and operational contexts. Artificial intelligence (AI) enhances automation, efficiency, and decision-making, reducing human error and leading to job displacement, ethical concerns, and over-reliance on technology, which may impact worker well-being. Cobotics (CO) improves workplace safety and productivity by enabling human–robot collaboration. Yet, challenges such as initial costs, ethical considerations, and a lack of flexibility raise concerns about worker adaptability and employment stability. Cyber–physical systems (CPSs) facilitate real-time monitoring, automation, and seamless integration, enhancing workplace safety, yet security risks, privacy concerns, and the need for specialized skills present new OSH challenges. The Internet of Things (IoT) improves connectivity, smart systems, and workplace security, but raises concerns regarding cybersecurity, data privacy, and system reliability, potentially exposing workers to new vulnerabilities. Big data (BD) enables predictive analytics, operational efficiency, and personalized work environments; however, issues related to data security, infrastructure requirements, and ethical considerations necessitate robust OSH frameworks to protect worker rights. Lastly, simulation offers a risk-free environment for training and decision support. Yet, the simplifications and assumptions inherent in simulations may lead to gaps between virtual training and real-world hazards. While digitalization and ICT drive significant advancements in work organization, they also introduce emerging OSH risks that require adaptive regulatory frameworks, workforce reskilling, and robust cybersecurity measures to ensure worker safety and well-being.

## 4. Discussion

This chapter discusses the key findings from the study, analyzing the impact of ICT and digitalization on occupational safety and health (OSH). It interprets the results in the context of the existing literature, highlights both the advantages and challenges of digitalization, and explores potential strategies for mitigating risks. Furthermore, this section examines the health implications of work-related exposure to ICT and the effectiveness of current regulatory frameworks, as well as proposes recommendations for future research and policy development.

### 4.1. The Positive and Negative Effects of ICT and Digitalization on the Workplace

Wallace [51] described that the advent of ICT has significantly transformed work and employment dynamics. It has led to new communication patterns, work practices, and blurred business boundaries. This shift has also resulted in a reduction in hierarchical structures [52]. The nature of work has undergone notable changes in this ICT-driven era. It has become more cognitively complex, team-based, reliant on social skills and technological competence, time-pressured, and mobile [53]. Researchers argue that employees in this new environment must possess kaleidoscope thinking abilities [54]. This refers to the capacity to consider different perspectives and integrate fragmented information into innovative patterns. Given these transformations, we must reassess our working environments [55], and rethink work processes and content [56]. Adapting to this changing landscape and embracing new approaches that support collaboration, flexibility, and the integration of diverse perspectives is crucial. By doing so, organizations can harness the potential of ICT-driven work environments and foster a culture of innovation.

However, the predictions regarding the changing nature of work and the emergence of knowledge-based societies and information and communication technology (ICT) have been around for quite some time. Initially, there was a waning interest among academics and practitioners as the actual progress did not align with the anticipated advancements. This changed more recently with the rapid proliferation of smart devices connected to the Internet. This resurgence has sparked renewed enthusiasm for the transformative potential of ICT in workplaces.

The predicted transformations are now being implemented quickly on a large scale. The knowledgefication, projectification, and virtualization of work are now prevalent across various domains and job types [57]. These changes yield outcomes and consequences that may not have been fully anticipated, such as jobless growth [58]. While ICT is rapidly progressing toward a Computer-Assisted Socially Mediated Information Technology (CASMIT) framework, the potential effects of ICT on work are still subject to divergent perspectives [59,60], as reflected in Table 6.

Adopting ICTs offers several positive aspects, including the potential to enhance workplace safety [70], and effectively mitigate and prevent occupational risks [71]. These technologies can be applied in various ways, such as utilizing industrial robots to exclude humans from hazardous environments [72], and continuously monitoring the workplace for factors like noise, temperature, and humidity to improve safety [73], promoting better industrial hygiene [74], employing smart devices to control machine safety advancements [75], fostering a safety-conscious culture and climate, and encouraging a proactive approach to occupational safety [76]. Moreover, ICT enables better occupational safety and health (OSH) management [8], facilitating improvements in OSH applications and practices to meet evolving requirements and standards [45].

Furthermore, the adoption of ICTs can contribute to enhancing worker safety in various ways. For instance, smart Personal Protective Equipment (PPE) can improve safety standards [45]. Continuous monitoring of vital worker health status and behavioral patterns, such as fatigue and insecurity, can help prevent accidents [41]. Research suggests that worker health may benefit from implementing virtual ergonomic analysis, which can reduce the incidence of musculoskeletal disorders [77].

Adopting ICTs can also have adverse effects, including increased psychosocial risks associated with the work context, organizational working styles, work-related suffering, and damage [8,73,78]. It can contribute to heightened stress levels [79] and mental fatigue [80]. Adopting these technologies may introduce new risks in the work environment, such as the risk of electric shock, risks associated with human–robot interaction, and the risk of cyber attacks [81]. Surveillance levels may decrease due to increased reliance on smart devices [79]. Furthermore, sedentary behavior resulting from reduced physical movements and activities can lead to health problems such as poor circulation and weakened bones and muscles [82].

### 4.2. Measures Taken to Tackle the Challenges and Optimize the Potential for Workplace Safety and Health

As mentioned in the preceding section, introducing ICT and digitalization will bring new and emerging challenges and opportunities to occupational safety and health (OSH). The extent to which these opportunities can be maximized depends on how technology is implemented, managed, and regulated. It is important to note that digital technology is neutral, with neither inherently positive nor negative qualities. A proactive and multi-faceted approach is required to maximize digitalization’s benefits while mitigating emerging occupational safety and health (OSH) risks. This includes ethical governance, worker-centered design, stakeholder collaboration, and regulatory adaptation. Implementing these strategies ensures that technology remains an enabler of workplace safety rather than a source of additional risks. Some potential measures include the following:

Ethical and Governance Frameworks: A robust ethical framework is essential to address the psychosocial and ergonomic challenges digitalization poses. As ICT and automation increasingly mediate workplace interactions, clear ethical guidelines should govern workplace surveillance, algorithmic management, and digital monitoring.

- Establishing Ethical Standards: Organizations should define ethical policies on digital workplace transformation, ensuring fairness and worker well-being [8]. Ethical guidelines should include policies on data privacy, algorithmic decision-making transparency, and limits on employee monitoring [83].
- Preventing Digital Overload: Companies must regulate work intensity by setting limits on digital communication, such as enforcing the “Right to Disconnect” policy [13]. Over-reliance on digital tools without proper regulatory oversight leads to work intensification, increased stress, and digital fatigue [84].
- Ensuring Cybersecurity and Privacy Protections: The increasing use of digital tools in OSH management raises concerns about data security. Organizations must ensure compliance with data protection regulations such as the General Data Protection Regulation (GDPR) to safeguard worker information [35].

Worker-Centered Design and Prevention Measures: Introducing new digital tools should prioritize human factors, integrating OSH measures to prevent negative health effects. The “prevention through design” approach is crucial in developing technologies that enhance safety rather than introduce new risks.

- Integrating Human Factors in Digital Workplaces: Technologies should be designed to reduce ergonomic risks, including musculoskeletal disorders from prolonged screen use and repetitive tasks [73]. Smart workplaces must consider lighting, posture, and workstation adjustability to optimize worker well-being.
- AI-Driven OSH Enhancements: Artificial intelligence (AI) can be leveraged for predictive safety measures, identifying hazards before they cause harm [71]. AI-enabled risk assessments and simulations provide real-time feedback, allowing organizations to adjust workflows accordingly [70].
- Smart PPE for Occupational Risk Prevention: Developing smart Personal Protective Equipment (PPE) can help mitigate workplace hazards. Wearable technology, such as biometric sensors, can monitor worker fatigue, exposure to harmful substances, and ergonomic stress [41].
- Occupational Stress Management Programs: Employers should implement well-being initiatives, including mental health support programs and digital detox strategies, to counteract the negative psychological effects of work digitalization [85].

Stakeholder Collaboration: Successfully implementing OSH strategies in a digitalized workplace requires collaboration among policymakers, industry leaders, labor unions, and researchers. A cooperative approach can ensure that technological advancements align with worker safety and well-being.

- Multi-Stakeholder Engagement: Governments, employers, and trade unions should work together to establish policies that protect workers in digital environments. The European Union’s “OSH overview” project highlights the need for collaborative efforts to address digitalization’s impact on OSH [86].
- Worker Participation in Digital Strategy Development: Employees should be actively involved in discussions about workplace digitalization. Organizations should develop participatory frameworks where workers can provide feedback on digital tools and risk management strategies [56].
- Academia–Industry Partnerships for OSH Innovation: Collaboration between researchers and industries can drive innovation in workplace safety. Research initiatives should focus on developing AI-driven OSH management systems, virtual ergonomic analysis, and digital risk prediction models [18].

Regulatory Adaptation and Policy Development: As digitalization accelerates, existing OSH regulations must evolve to address emerging workplace risks. Policymakers should update labor laws and safety standards to reflect the realities of Industry 4.0.

- Expanding Legal Protections for Digital Workers: Policies such as the “Right to Disconnect” should be universally enforced to prevent excessive work-related stress and maintain a balance between professional and personal life [83].
- Standardizing AI and Automation in OSH Management: Governments should create legal frameworks that regulate AI-driven workplace safety systems. Algorithmic decision-making in workforce management must be transparent, fair, and aligned with ethical labor practices [35].
- Establishing New OSH Standards for Digital Workspaces: Existing workplace safety standards should be updated to include guidelines for remote work, hybrid workplaces, and automated safety monitoring systems [13].

Training and Reskilling the Workforce: One of the most critical elements of optimizing OSH benefits is ensuring workers are equipped with the necessary skills to navigate digital work environments safely.

- Developing Digital Literacy Programs: Workers should be trained to use new technologies efficiently while understanding potential risks [31]. Digital literacy programs can help employees manage digital workload, cybersecurity threats, and ergonomic best practices.
- Upskilling for Emerging Digital Roles: Training programs should focus on reskilling workers for new job functions that arise from automation and AI integration [39]. AI-assisted training tools and virtual simulations can help workers adapt to Industry 4.0 environments.
- Continuous Learning and Occupational Health Awareness: Organizations should implement ongoing occupational safety training, ensuring employees are aware of traditional and digital workplace hazards [48].

To ensure that digitalization enhances workplace safety rather than introduces new risks, a combination of ethical governance, technological design improvements, collaborative policymaking, and continuous worker education is required. Organizations must proactively address the psychological, ergonomic, and cybersecurity risks associated with ICT adoption while leveraging smart technologies to optimize OSH outcomes. A worker-centric approach ensures that technological advancements contribute to healthier, safer work environments.

### 4.3. The Health Impacts of Work-Related Exposure to ICT and Digitalization

Digitalization has been a subject of intense debate in political and economic discussions, with differing views on whether it is a problem or a solution for work life. However, considering users’ perspectives, exposure to information and communication technology (ICT) can demand high social, physical, and cognitive skills [87,88]. This could lead to stress among users [89], with stress being perceived as a negative consequence of ICT use. Previous research has identified connections between ICT demands and cognitive impairments [90], poor self-reported health [91], and even burnout related to ICT use [85]. The term “technostress” has become widely used across fields like information systems and psychology to describe the health impacts of ICT use in the workplace. First introduced by Brod (1982) [92], it highlighted the growing disconnect between individuals and computers, particularly the lack of psychological adjustment. In this paper, we use the broader and more neutral term “ICT exposure” to refer to what has previously been called technostress, digital stress, or ICT demands in other research.

In certain situations, higher job demands may not necessarily harm workers’ health if they are supported by adequate resources, as suggested by the job demands–resources theory [93]. Studies have emphasized the importance of resources in mitigating potential negative effects, including the role of social support and support related to ICT [94]. Lazarus’ transactional theory of stress [95], a key concept in organizational psychology, helps explain when ICT exposure may lead to negative outcomes. This theory was applied to explore the health effects of ICT exposure within technostress [96]. It highlights how stress results from challenging conditions, known as “stressors”, and an individual’s response to them can lead to adverse outcomes or “strain”. Organizational factors that shape working conditions can determine whether ICT use is perceived as a source of strain. According to an EU foresight study EU-OSHA [97], psychosocial and organizational factors will play an increasingly significant role in occupational health as work becomes more digitalized. Digitalization may introduce changes that elevate the risk of stress, such as increased monitoring, expectations of constant availability, and the use of algorithms to manage work and workers.

Further exploration is needed to develop a comprehensive understanding of the circumstances in which ICT use can be viewed as an opportunity or a concern, as well as its potential adverse effects on health. Existing studies on occupational ICT exposure have primarily focused on highly qualified occupations despite the wide range of ICT usage patterns within the working population. A recent systematic review emphasized the importance of distinguishing between various occupational characteristics and found that few studies adequately identified the organizational factors influencing ICT-related work processes [85]. These organizational factors can shape how ICT exposure affects workers, potentially leading to longer working hours, more significant work–life interference, or increased work intensification [84]. Work intensification, a key trend in today’s digital workplaces, continues to influence the outcomes of ICT use, a phenomenon known as digital work intensification [98,99]. Chesley (2014) conducted a study that explored how ICT use contributes to employee strain and distress, finding that higher levels of strain and distress are associated with work intensification driven by ICT. Therefore, it is crucial to distinguish between ICT use and digital work intensification to assess whether broader organizational factors influence the health effects of ICT use [100].

### 4.4. ICT and Digitalization Legislative and Regulatory Frameworks

The transformative impact of the digital revolution on production processes and value creation is profound, posing significant challenges for businesses. To stay competitive, companies must proactively develop strategies to harness the potential of digitalization, enhance existing processes, and innovate new business models [101]. It is crucial to consider the legal implications associated with these changes.

Although no specific European legislation focuses solely on ICT and digitalization, existing legal frameworks related to working conditions can be applied to these evolving work practices. Relevant examples include the Work-Life Balance Directive, the Working Time Directive, the European Framework Directive on Safety and Health at Work, and the Transparent and Predictable Working Conditions Directive. Additionally, various EU initiatives and policies aim to reduce the digital divide, ensure equal access to telework, and address regional inequalities. The European Social Partners have also developed framework agreements on telework (2002) and digitalization (2020), which address various ICT-related issues [83].

Several EU Member States have implemented policies and laws to address the challenges associated with ICT-based telework and mobile work. However, the approaches taken by individual countries vary significantly due to differences in institutional structures, legislative frameworks, industrial relations systems, cultural contexts, and levels of digital development. Most Member States (21 out of 27) have enacted legislation specifically targeting ICT work or regulating certain aspects. In some Scandinavian countries, the regulation of ICT, telework, and mobile work is primarily left to collective bargaining. In contrast, other countries, like Ireland, have opted for more flexible measures such as codes of conduct or guidelines [83].

Despite these disparities, collective agreements and practices in large companies serve as the primary mechanisms influencing the practical utilization of ICT across most EU nations. The “right to disconnect” has recently gained prominence in legislation, collective agreements, and company practices. This concept aims to counteract the adverse effects of ICT work and safeguard employees’ non-working time.

From 2020 to 2022, EU-OSHA undertook an “OSH overview” project aimed at offering valuable insights into the policy, prevention, and practices concerning the challenges and opportunities arising from digitalization in the field of occupational safety and health (OSH) [86]. This overview of occupational safety and health (OSH) expands on insights from the foresight study on digitalization and OSH and key findings from the third edition of EU-OSHA’s European Survey of Enterprises on New and Emerging Risks. The focus is understanding how digitalization shapes workplace risks and safety measures across European Union enterprises. The OSH overview encompasses various projects that utilize literature reviews, surveys, interviews, case studies, and policy/practice evaluations. This overview addresses several critical areas, including the effects of task automation and changes in job content on occupational safety and health (OSH), the role of smart, collaborative robotics (cobots) and worker monitoring about OSH, and the management of workers through AI or algorithms, such as in gamified work environments and online platform work. It also provides updates on EU-OSHA’s regulatory and policy advancements, alongside qualitative and quantitative research on OSH issues that workers face in online platforms. Furthermore, the overview features case studies showcasing effective OSH practices in the digital workplace, highlighting technologies like Virtual Reality (VR), Augmented Reality (AR), and smart Personal Protective Equipment (PPE), contributing valuable insights to the Healthy Workplaces Campaign on Digitalization.

### 4.5. Theoretical Foundations of Digitalization and Occupational Safety and Health

The rapid digitalization of workplaces presents opportunities and challenges for occupational safety and health (OSH). While digital technologies improve efficiency, safety monitoring, and risk assessment, they also introduce long-term concerns, such as job displacement, technostress, and evolving human–machine interactions. Understanding these implications requires a robust theoretical foundation that connects digitalization to existing frameworks in labor economics, psychology, and sociotechnical systems. This section expands the theoretical context by incorporating automation theories, the Technology Acceptance Model (TAM), sociotechnical systems theory, and labor economics frameworks to provide a deeper academic foundation for the study.

#### 4.5.1. Automation Theories and the Future of Work

The increasing integration of automation, artificial intelligence (AI), and robotics in the workplace aligns with established automation theories examining labor market shifts and job structures. According to [102], automation threatens routine-based occupations, potentially leading to large-scale job displacement. However, [103] argues that technological advances create new employment opportunities by complementing human capabilities rather than fully replacing them. These perspectives are crucial in analyzing how digitalization influences OSH, particularly regarding workforce transformation, job security, and new workplace risks associated with human–robot collaboration.

#### 4.5.2. Technology Acceptance Model (TAM) and Digital Work Adaptation

Davis’ Technology Acceptance Model [104] provides a valuable framework for understanding how workers and organizations adapt to digitalization and how the ease of use of digital systems influences their adoption rates. In the context of OSH, digital tools such as AI-driven risk assessment software, wearable safety devices, and remote monitoring systems are only effective if employees perceive them as beneficial and easy to integrate into daily workflows. Resistance to digital transformation, often due to concerns over surveillance or job insecurity, highlights the importance of designing workplace technologies prioritizing worker well-being and autonomy.

#### 4.5.3. Sociotechnical Systems Theory and Worker–Technology Interaction

Sociotechnical systems theory emphasizes the interaction among people, technology, and organizational structures. According to [105], technological advancements must be aligned with social and human factors to create sustainable work environments. In digitalized workplaces, poorly integrated systems can lead to increased stress, work intensification, and ergonomic challenges. By applying sociotechnical principles, organizations can develop human-centered digital work environments that balance efficiency with worker well-being, ensuring that digitalization enhances OSH rather than introducing new hazards.

#### 4.5.4. Labor Economics and the Changing Nature of Work

The evolution of digital workplaces can also be analyzed through labor economics frameworks, particularly the Skill-Biased Technological Change (SBTC) theory. According to SBTC, technological advancements disproportionately benefit high-skilled workers while reducing opportunities for low-skilled labor [106]. This trend has direct OSH implications, as low-skilled workers may face greater job insecurity, increased work surveillance, and pressure to adapt to digital workflows without adequate training. Policymakers must address these disparities by implementing workforce reskilling programs and ensuring that digitalization does not exacerbate inequalities in workplace safety and well-being.

By integrating automation theories, TAM, sociotechnical systems theory, and labor economics, this study establishes a stronger theoretical foundation for analyzing digitalization’s impact on OSH. These frameworks provide insights into how workers adapt to technological changes, the risks associated with automation, and strategies for designing human-centered digital workplaces. Strengthening the connection between digitalization and existing theories enhances the academic contribution of this research, offering a comprehensive understanding of both the short-term and long-term implications of workplace digital transformation.

### 4.6. Recommendations

The literature review has revealed that ICT and digitalization, along with their components, have both positive and negative effects on occupational safety and health (OSH). While digital technologies enhance workplace efficiency, safety, and real-time monitoring, they also introduce new risks, such as psychosocial stressors, ergonomic challenges, and cybersecurity threats.

During this transitional period, one significant challenge is the inadequacy of existing OSH initiatives, including regulatory frameworks and safety standards, which often struggle to keep pace with evolving ICT-driven risks. Furthermore, there is a risk of losing the proactive approach to OSH that has been established in many industrialized nations [8,73]. As digitalization reshapes workplaces, various researchers and organizations, including the European Factories of the Future Research Association [107], emphasize the urgent need for proactive, adaptable, and interdisciplinary approaches to safeguard worker well-being.

Targeted policy interventions and research initiatives are necessary to address these challenges. This section provides concrete, stakeholder-specific recommendations for policymakers, organizations, and researchers to ensure that digitalization enhances OSH without compromising worker well-being.

**Recommendations for Policymakers:** Policymakers play a crucial role in shaping regulatory frameworks to ensure worker protection in digital workplaces. The following actionable measures should be considered:

I. Strengthening Legal Protections for Digital Workers
  - Expand and enforce the “Right to Disconnect” legislation [13] across industries to address the risks of work intensification and burnout in digital environments. Member States should mandate clear disconnection policies and impose penalties for non-compliance.
  - Introduce legal standards for AI-driven workforce management to ensure transparency, fairness, and worker autonomy. Algorithmic decision-making must be regulated to prevent excessive monitoring and bias in performance evaluations [35].
  - Update OSH directives to include digital workplace risks, such as technostress, cybersecurity vulnerabilities, and AI-mediated labor relations. The European Agency for Safety and Health at Work (EU-OSHA) should integrate these aspects into existing occupational safety guidelines [86].
II. Establishing Industry-Specific OSH Guidelines
  - Develop sector-specific digital safety standards to address unique risks in industries such as manufacturing, healthcare, and remote work environments [71]. Regulations should include AI-integrated risk assessments, smart PPE requirements, and human–robot interaction protocols.
  - Mandate employer-provided OSH training programs on digital work safety to ensure workers are adequately prepared for technology-driven workplaces [39].

**Recommendations for Governments**

I. Strengthening and expanding the “Right to Disconnect” Laws
  - Governments should enact and enforce clear legislation ensuring workers have the legal right to disengage from work-related communications outside of contractual hours. The EU’s “Right to Disconnect” policy was implemented in countries such as France, Spain, and Germany, reducing work-related stress and burnout [108]. However, enforcement remains inconsistent across industries. Governments should introduce mandatory compliance audits and penalties for non-adherence.
  - Lessons from France show that while the law has improved work–life balance, employer resistance and lack of awareness have limited its effectiveness. Governments should introduce awareness campaigns and employer training programs to enhance compliance.
  - Countries without such laws should establish legislative frameworks requiring employers to provide clear disconnection policies modeled after successful implementations in the EU.
II. Regulating AI-Driven Workplace Monitoring and Algorithmic Decision-Making
  - Governments must introduce algorithmic transparency laws requiring employers to disclose how AI-driven decisions impact employment conditions. Workers should have the right to contest unfair algorithmic evaluations.
  - National labor agencies should develop guidelines for ethical AI implementation to prevent excessive surveillance, ensure data privacy, and uphold worker autonomy [109].
III. Developing Industry-Specific OSH Standards for Digital Work Environments
  - OSH regulations should be updated to address technostress, cybersecurity vulnerabilities, and AI-mediated labor relations.
  - Governments should create sector-specific digital safety standards, ensuring that industries with high automation levels (e.g., manufacturing, healthcare) integrate smart PPE, AI-driven hazard detection, and fatigue monitoring systems into their safety protocols.

**Recommendations for Labor Unions and Worker Organizations**

I. Negotiating Stronger Protections for Digital Workers
  - Labor unions should push for collective bargaining agreements that include digital labor rights, ensuring the protection of work–life balance in AI-driven workplaces [110].
  - Unions must advocate for transparent AI governance, requiring companies to disclose the criteria used in AI-based performance evaluations.
II. Educating Workers on Digital Labor Rights
  - Unions should conduct awareness campaigns on the “Right to Disconnect” and other labor protections, ensuring workers know their rights in digitalized work environments.
  - Collaboration with policymakers is essential to ensure that digital workers receive adequate training on AI, cybersecurity, and digital OSH measures.

**Recommendations for Businesses, Organizations, and Employers**: Employers must proactively implement safety measures that align with evolving OSH risks in digital workplaces. The following concrete actions can enhance workplace safety and well-being:

I. Implementing Structured “Right to Disconnect” Policies
  - To prevent overwork, businesses should establish company-wide policies defining permissible working hours and communication expectations.
  - Employers must ensure that disconnecting from work is not penalized and should integrate this policy into employment contracts and HR guidelines.
  - Develop clear company-wide “Right to Disconnect” policies, defining permissible working hours and communication expectations to prevent overwork [83].
  - Adopt AI-driven OSH monitoring tools that balance efficiency and worker autonomy. AI applications should enhance safety (e.g., fatigue detection) rather than serve as intrusive surveillance mechanisms [55].
II. Investing in Employee Well-being and Training
  - Integrate mandatory OSH digital literacy programs into employee training to enhance cybersecurity awareness and safe technology use [31].
  - Promote workplace ergonomics by incorporating smart workstations equipped with posture correction, blue-light reduction, and adjustable seating features to prevent digital-related physical strain [73].
  - Provide continuous access to mental health and stress management programs to counteract digital fatigue, burnout, and technostress [85].
III. Enhancing Workplace Ergonomics for Digital and Hybrid Workforces
  - Organizations should implement ergonomic workplace solutions, including adjustable workstations, blue-light-reducing screens, and AI-powered fatigue monitoring to prevent digital-related physical strain.
  - Employers should invest in mental health programs, ensuring employees can access digital detox strategies and workplace counseling.

**Recommendations for Researchers and Academia**: Further research is essential to explore the long-term impacts of digitalization on OSH and develop evidence-based interventions. Key research priorities include the following:

I. Investigating the Long-Term Effects of Digital Work on Health
  - Conduct longitudinal studies on digital work intensification to assess its impact on mental health, sleep quality, and overall well-being [91].
  - Examine the relationship between automation and occupational safety, with a focus on emerging risks from human–robot collaboration and AI-driven decision-making [45].
II. Developing Smart Safety Technologies and Solutions
  - Advance research on AI-driven OSH management systems, integrating real-time hazard detection and worker fatigue monitoring [70].
  - Investigate the reliability of smart PPE and wearable safety devices in high-risk environments such as construction and manufacturing [41].
III. Policy-Oriented Research on Digital Labor Regulations
  - Analyze the effectiveness of existing OSH laws in digital work environments and propose updates to labor policies based on empirical data [86].
  - Assess the role of collective bargaining and social dialog in shaping fair and inclusive digital workplaces.

Table 7 summarizes the actionable recommendations for each key stakeholder group—policymakers, organizations, and researchers—to improve occupational safety and health (OSH) in the digitalized workplace.

By implementing these targeted strategies, stakeholders can enhance occupational safety and health standards in digitally evolving workplaces while minimizing emerging risks. Future research should explore innovative safety technologies and evidence-based policy frameworks to ensure that the integration of digitalization and ICT continues to support, rather than undermine, worker well-being.

### 4.7. Study Limitations and Future Work

While this study provides valuable insights into the impact of digitalization and ICT on occupational safety and health (OSH), several limitations must be acknowledged. These limitations affect the findings’ validity, generalizability, and applicability, particularly in evolving workplace technologies. Addressing these challenges is essential to comprehensively understanding the subject matter.

One of the primary limitations of this study is its reliance on literature sourced exclusively from the Scopus database. While Scopus is a well regarded academic resource, it does not encompass all relevant publications, particularly those found in non-indexed journals, industry reports, and gray literature. This selection introduces the potential for publication bias, as high-impact journals dominate the discourse, potentially overlooking valuable industry perspectives and practical case studies. As a result, the study may lean toward theoretical and well-documented academic findings rather than incorporating real-world applications of ICT in occupational safety. Future research should aim to expand database coverage by including sources such as Web of Science, IEEE Xplore, Google Scholar, and policy reports from international organizations like the International Labour Organization (ILO) and the European Agency for Safety and Health at Work (EU-OSHA). A broader range of sources would help mitigate publication bias and provide a more holistic view of the topic.

Another significant limitation arises from the exclusion of non-English language publications. By focusing only on English language studies, this research may have inadvertently overlooked valuable insights from non-English-speaking regions where digitalization in the workplace may differ substantially from Western contexts. Countries such as China, Japan, Brazil, and Germany have unique approaches to integrating digital technologies into occupational safety frameworks, yet their perspectives remain underrepresented in this study. The exclusion of these sources limits the study’s ability to capture the full global impact of ICT on OSH. To overcome this, future research should incorporate multilingual sources or collaborate with researchers proficient in other languages to ensure a more inclusive analysis of workplace digitalization trends.

Additionally, the study’s methodology was based on a keyword-driven search strategy, which, while systematic, may have constrained the scope of the literature review. Different disciplines often use varying terminologies to describe similar concepts. For instance, studies from computer science may frame workplace automation differently than those from occupational psychology or labor economics. As a result, relevant research that employs alternative terminology may have been omitted, potentially leading to gaps in the reviewed literature. Future systematic reviews should incorporate broader and more flexible search strategies to address this limitation, utilizing techniques such as synonym expansion and natural language processing (NLP) to capture a wider range of studies across multiple disciplines.

Moreover, this study is largely theoretical, relying on a literature review rather than empirical data collection. While reviewing existing studies provides a strong foundation for understanding ICT’s role in workplace safety, it does not capture real-world experiences of workers, employers, or policymakers. Without empirical validation, it is difficult to determine the practical applicability of the findings or assess how digitalization affects workers in diverse job sectors. To enhance the robustness of future research, empirical studies employing surveys, workplace observations, interviews, and longitudinal research should be conducted. Such approaches would allow for a more nuanced understanding of how ICT impacts OSH across different industries and employment settings.

Furthermore, while this study identifies regulatory challenges associated with digitalization, it does not conduct a comparative analysis of how different countries implement and enforce digital workplace policies. Digital labor policies vary significantly across regions, and some nations have been more proactive in regulating AI-driven workforce management, worker surveillance, and automation safety protocols. For example, Scandinavian countries have established progressive labor policies balancing automation and worker protections, while Japan has pioneered regulations addressing human–robot collaboration risks. By not examining these international best practices in detail, the study misses an opportunity to identify scalable policy solutions that could be adapted across various labor markets. Future research should include cross-country policy analyses to compare regulatory effectiveness and explore best practices in managing digital workplace transformations.

Several key areas warrant further investigation to build on these findings and address the identified limitations. First, empirical research should be prioritized to collect firsthand data on how digitalization affects workplace safety, well-being, and regulatory compliance. Surveys and case studies could provide insights into sector-specific differences, while longitudinal studies could track the long-term impact of ICT on employee health and productivity. Second, quantitative methods should be employed to analyze extensive datasets and ascertain measurable correlations between digitalization and OSH outcomes. Machine learning and statistical modeling could be instrumental in identifying patterns of digital work stress, accident rates, and safety improvements resulting from automation.

Another crucial area for future research involves investigating the ethical and legal implications of AI-driven workplace safety. The increasing reliance on algorithmic decision-making in workforce management raises concerns about worker autonomy, data privacy, and algorithmic bias. Research should explore how AI governance frameworks can be improved to ensure fairness and transparency in digital work environments. Additionally, the effectiveness of existing labor laws in mitigating digital work risks should be assessed, focusing on policies such as the European Union’s “Right to Disconnect” and the regulation of AI in occupational safety monitoring.

Finally, cross-cultural and comparative studies on digital labor policies should be conducted to identify successful regulatory frameworks. Examining how different countries manage the integration of digital technologies in workplace safety can offer valuable insights into best practices that can be adopted internationally. Research should also explore the role of trade unions and worker advocacy groups in shaping fair and equitable digital workplaces.

In conclusion, while this study provides a foundational analysis of the intersection among ICT, digitalization, and occupational health and safety, its limitations highlight the need for further empirical, interdisciplinary, and policy-focused research. Addressing publication bias, expanding language inclusivity, refining search methodologies, and incorporating real-world data will enhance the validity and applicability of future findings. As workplace digitalization continues to evolve, ongoing research will be critical in ensuring that technological advancements contribute to safer, healthier, and more sustainable work environments.

## 5. Conclusions

The primary aim of this paper was to offer dependable and high-quality information regarding the emerging occupational health and safety (OSH) risks associated with digitalization, changes in information and communication technology (ICT), and their influence on work, encompassing both positive and negative aspects. A literature review effectively addressed the research questions, providing insights into established connections and suggesting potential actions in linking ICT, digitalization, and OSH. This contribution significantly enhances the existing literature on the subject matter.

A total of 50 documents were examined to analyze the impact of adopting ICTs on occupational safety and health (OSH). This comprehensive review enabled the identification and categorization of the effects as positive or negative, enhancing understanding in this area. Although the number of publications remains relatively limited, there has been significant and rapid growth, indicating the current research interest in this field. It is important to note that studies in this domain are still in the exploratory stage, and numerous questions regarding the relationship among ICT, digitalization, and OSH still need to be answered. These observations align with the findings of other authors [8,73,76,79].

In terms of theoretical implications, this study offers the following contributions: (i) the identification of key technologies relevant to occupational health and safety (OHS); (ii) the identification of positive impacts of technologies on OSH; (iii) the identification of negative effects of technologies on OSH, which may increase risks to the environment and worker safety; and the (iv) identification of research limitations and suggestions for future work, highlighting the need for further investigation, particularly in understanding the impacts of ICT and digitalization on workers and the work environment.

The technologies explored in this study present several advantages, such as providing valuable data for analysis and decision-making, enhancing workplace conditions and employee well-being monitoring, and ultimately supporting occupational safety and health (OSH). However, it is crucial to recognize that these technologies may also introduce new risks, mainly when information overloads. If workers are not sufficiently informed about the purpose of data monitoring or cannot perceive clear benefits from these technologies, it may lead to increased stress levels.

## Figures and Tables

**Table 1:** Filter criteria.

Section	Description
Criterion 1	Book, book series, trade journal, conference proceeding, erratum, editorial, review
Criterion 2	Studies which are not in English
Criterion 3	We excluded publications lacking the search terms “health and safety”, “Industry 4.0”, “smart technologies”, “smart manufacturing”, “smart industry”, or “CPS” in the title, abstract, or keywords
Criterion 4	Studies in press
Criterion 5	Publications that failed to discuss ICT and digitalization concerning OSH were omitted

**Table 2:** The technological domains associated with ICT and digitalization of the workplace.

Category	Definition
Big data	Massive and intricate datasets that surpass the capabilities of conventional data processing techniques to manage, analyze, or interpret effectively. These datasets are typically defined by four key attributes: volume (scale of data), velocity (speed of data generation and processing), variety (different types and sources of data), and veracity (accuracy and reliability of the data) [20].
Internet of Things	A network of physical objects, or “things”, is equipped with software, sensors, and connectivity features that allow them to gather and share data via the Internet [21].
Cyber–physical system	A tightly integrated system that merges computational (cyber) elements with physical components, resulting in intelligent, interconnected networks capable of real-time monitoring, analysis, and control of physical processes [22].
Cobotics	A developing field of technology focusing on designing robots by integrating disciplines such as information sciences, human factors (including behavior, decision-making, robustness, and error monitoring), biomechanics (including modeling of behavior and movement dynamics), and robotics [23].
Artificial intelligence	Integrating various disciplines, theories, techniques, concepts, and technologies to create machines that can simulate intelligence [24].
Simulation	The representation of the behavior of an industrial process using a computer model, where the parameters and variables of the model correspond to those of the actual process being investigated [25].

**Table 3:** A clear overview of the primary journals contributing to the research area (journals with more than two publications).

Journal Name	Number of Articles
*Sustainability Switzerland*	4
*Computers and Industrial Engineering*	3
*International Journal Of Advanced Manufacturing Technology*	2
*IEEE Access*	2
*American Journal of Industrial Medicine*	2
*Safety Science*	2

**Table 4:** Some of the selected publications.

Review Article	Technological Categories	Keywords
[27]	IOT, AR, VR, AI	Industry 4.0; exposure assessment; safety; air quality; occupational health and safety
[28]	Internet of Things (sensors)	Industry 4.0; Manufacturing machines; Sensors; Occupational safety
[29]	Sensors, AI	Human activity recognition; pressure insoles; explainable AI; Industry 4.0
[30]	CPS, AI, cloud computing, AR, VR, Big data	Construction 4.0; economic sustainability; environmental sustainability; Industry 4.0; social sustainability; sustainability framework; United Arab Emirates (UAE)
[31]	Cobots	Human–robot Collaboration; Industry 4.0; Occupational Safety; Static and dynamic analysis
[32]	Cloud Computing	Digital industry; Digital workers; E-mental health; Intention; Mental health literacy; Workplace wellness
[33]	Smart cities	artisanal and small-scale mining; Rwanda; awareness of 3T minerals; SMART technologies; socioeconomic impact
[26]	VR, BD, IoT, AI, CPS, Robotics, Simulation.	The Fourth Industrial Revolution; Safety; Occupational health and safety; Systematic literature review
[34]	IoT, Smart PPE	Industry 4.0; occupational risk assessment; internet of things; smart PPE
[35]	Robotic, AI	AI-based systems; human factors; human in control; ICT; occupational safety and health (OSH); robotic systems
[36]	IoT	education; occupational risk; teacher; technostress
[37]	Smart Cyber–Physical	digitalization of OSH; OSH 4.0 policy and strategy; OSH 4.0; OSH 5.0 model; socio-technical systems safety science
[38]	Cobots, Exoskeletons, IoT	Aging workforce; Digitalization; Productivity; Safety; Technologies
[39]	IoT	Human-centered Industry; Industry 4.0; Internet of Things; Occupational Health and Safety; Occupational Well-being
[40]	CPS	BCI applications; BCI challenges; EEG; Industry 4.0; technology transfer
[41]	Big Data, IoT	Communications; Data collection; IIoT; Localization; Occupational safety; Smart devices; Wearables
[42]	CPS, IoT, Robotics	Ergonomics; Methodological framework; Mocap system; Occupational Safety; Operator 4.0
[43]	AI, IoT, AR, VR	Education; eLearning; Human Augmentation; Mixed Reality; Virtual Reality
[44]	AI, Cobots	Artificial intelligence; ICT; industrial robot; semantic model
[45]	BD, VR, IoT, AI, CPS, Robotics, Simulation.	digital factory; OSH 4.0; indoor positioning systems; Industry 4.0
[46]	CPS, AI	Flexible work arrangements; Mobile work; OSH; Institutional health and safety systems; Occupational health and safety
[47]	CPS, AI, IoT	Digitalization; Integration; Safety
[48]	CPS, sensors	Accident prevention; Industry 4.0; Occupational risks;
[8]	VR, BD, IoT, AI, CPS, Robotics, Simulation.	Occupational safety and health (OSH); Industry 4.0
[49]	AR, VR, IoT	Antifragility engineering; Augmented reality; Industry 4.0; Occupational safety; Resilience engineering; Virtual reality

ICT and digitalization technologies: CPS: Cyber–physical system; CC: cloud computing; AI: artificial intelligence; AR: Augmented Reality; BD: big data; IoT: Internet of Things; RO: robotics; SM: simulation; VR: Virtual Reality.

**Table 5:** ICTs.

Category	Advantages	Disadvantages
Artificial Intelligence (AI)	Automation and Efficiency Efficient Decision-making and Problem-solving Improved Accuracy	Job Displacement Ethical Concerns Lack of Creativity and Intuition Dependency and Reliability
Cobotics (CO)	Increased Efficiency and Productivity Improved Safety Enhanced Accuracy and Quality Cost Savings	Job Displacement Initial Cost and Maintenance Lack of Flexibility Ethical Considerations Dependency and Reliability
Cyber–Physical Systems (CPSs)	Improved Efficiency Enhanced Automation Real-Time Monitoring and Control Interconnectivity and Integration Adaptability and Flexibility	Security Risks Complexity and Integration Challenges Privacy Concerns Skill and Knowledge Gap
Internet of Things (IoT)	Enhanced Connectivity and Communication Smart and Intelligent Systems Improved Safety and Security Enhanced Quality of Life	Privacy and Security Concerns Interoperability and Compatibility Issues Scalability and Complexity Reliability and Dependence on Connectivity
Big Data (BD)	Enhanced Decision-Making Improved Operational Efficiency Personalization and Customer Experience Innovations and New Business Opportunities Predictive Analytics and Forecasting	Data Security and Privacy Risks Data Quality and Reliability Infrastructure and Resource Requirements Ethical Considerations Skill Gap and Data Management
Simulation	Risk-Free Environment Performance Optimization Training and Skill Development Decision Support	Simplification and Assumptions Limited Realism Data Requirements Model Verification and Validation

**Table 6:** Overview of ICT impacts on work.

Reference	Aspects of Job Quality	Positive	Negative
[61]	Employment	Developing new markets and investing in human capital lead to the creation of new job opportunities.	Automation and rationalization lead to the destruction of jobs.
[30]	Employment Relations	As knowledge-based work expands, trust levels rise.	Trust levels decrease when workers are constantly monitored in real-time using information and communication technology (ICT).
[62]	Career	New possibilities for boundaryless careers arise.	Unexpected competition disrupts the traditional progression of career ladders.
[63]	Job Prospects	Connected and open organizations enhance career opportunities and expand them.	The presence of surveillance and outsourcing leads to a rise in the number of “dead-end” jobs.
[64]	Skills	Workers are upgraded to handle multi-tasking and engage in creative, knowledge-based tasks and jobs.	Downgraded to performing personal services and tasks related to tending machines.
[65]	Workplace Relations	Individuals are interconnected and stimulated.	Workers are subjected to isolation, and experience heightened stress levels.
[66]	Life Balance	Work is seamlessly integrated and harmonized with everyday life.	Work permeates every aspect of life, regardless of time or location.
[67]	Work Intensity	Opportunities are presented to decrease work efforts through the assistance of machines.	This leads to an intensification of work and individuals taking on multiple jobs.
[68]	Compensate/Pay	People with specialized skills and expertise in knowledge-based fields often see increased earnings.	Pay is diminished as skills are downgraded, and individuals have limited bargaining power.
[69]	Social Structure	There is an increase in individual flexibility and the ability to make independent choices.	Society becomes more divided as power and control become centralized.

Adopted from [60].

**Table 7:** Summary of key recommendations.

Stakeholders Group	Actionable Recommendations
Policymakers	Strengthen “Right to Disconnect” laws, regulate AI in workforce management, and update OSH directives for digital workplaces.
Businesses/Organizations	To prevent overwork, implement clear work–life balance policies, adopt AI for safety monitoring, enhance digital literacy training, and improve workplace ergonomics.
Researchers	Conduct long-term health studies, develop AI-driven OSH solutions, and investigate policy gaps in digital labor regulations.
Labor Union	Push for collective bargaining agreements, advocate for transparent AI governance, conduct awareness campaigns, and collaborate with policymakers.

## Data Availability

Not applicable.

## References

[1] Peña-Casas R., Ghailani D., Coster S. (2018). The Impact of Digitalisation on Job Quality in European Public Services the Case of Homecare and Employment Service Workers.

[2] Schwab K. (2017). The Fourth Industrial Revolution.

[3] Adams A. (2018). Technology and the Labour Market: The Assessment. Oxf. Rev. Econ. Policy.

[4] Gontareva I., Chorna M., Pawliszczy D., Barna M., Dorokhov O., Osinska O. (2018). Features of the Entrepreneurship Development in Digital Economy. TEM J..

[5] Benson C., Obasi I.C., Akinwande D.V., Ile C. (2024). The Impact of Interventions on Health, Safety and Environment in the Process Industry. Heliyon.

[6] Bogle I.D.L. (2017). A Perspective on Smart Process Manufacturing Research Challenges for Process Systems Engineers. Engineering.

[7] Obasi I.C., Benson C. (2023). Evaluating the Effectiveness of Machine Learning Techniques in Forecasting the Severity of Traffic Accidents. Heliyon.

[8] Badri A., Boudreau-Trudel B., Souissi A.S. (2018). Occupational Health and Safety in the Industry 4.0 Era: A Cause for Major Concern?. Saf. Sci..

[9] Jamwal A., Agrawal R., Sharma M., Giallanza A. (2021). Industry 4.0 Technologies for Manufacturing Sustainability: A Systematic Review and Future Research Directions. Appl. Sci..

[10] Holtgrewe U. (2014). New New Technologies: The Future and the Present of Work in Information and Communication Technology. New Technol. Work Employ..

[11] Mazmanian M., Orlikowski W.J., Yates J. (2013). The Autonomy Paradox: The Implications of Mobile Email Devices for Knowledge Professionals. Organ. Sci..

[12] Wajcman J. (2016). ANFÖRANDE: Pressed for Time: The Digital Transformation of Everyday Life: Huvudanförande Vid Sociologidagarna i Uppsala 10–12 Mars 2016. Sociol. Forsk..

[13] Elhusseiny H.M., Crispim J. (2022). SMEs, Barriers and Opportunities on Adopting Industry 4.0: A Review. Procedia Comput. Sci..

[14] Fawcett S.E., Waller M.A., Miller J.W., Schwieterman M.A., Hazen B.T., Overstreet R.E. (2014). A Trail Guide to Publishing Success: Tips on Writing Influential Conceptual, Qualitative, and Survey Research. J Bus. Logist..

[15] Conforto E.C., Amaral D.C., Silva S. (2011). Da Roteiro Para Revisão Bibliográfica Sistemática: Aplicação No Desenvolvimento de Produtos e Gerenciamento de Projetos. Trab. Apresentado.

[16] Liberati A., Altman D.G., Tetzlaff J., Mulrow C., Gøtzsche P.C., Ioannidis J.P.A., Clarke M., Devereaux P.J., Kleijnen J., Moher D. (2009). The PRISMA Statement for Reporting Systematic Reviews and Meta-Analyses of Studies That Evaluate Health Care Interventions: Explanation and Elaboration. Ann. Intern Med..

[17] Duarte J., Rodrigues F., Castelo Branco J. (2022). Sensing Technology Applications in the Mining Industry—A Systematic Review. Int. J. Environ. Res. Public Health.

[18] Gualtieri L., Rauch E., Vidoni R. (2021). Emerging Research Fields in Safety and Ergonomics in Industrial Collaborative Robotics: A Systematic Literature Review. Robot. Comput.-Integr. Manuf..

[19] Chadegani A.A., Salehi H., Yunus M.M., Farhadi H., Fooladi M., Farhadi M., Ebrahim N.A. (2013). A Comparison between Two Main Academic Literature Collections: Web of Science and Scopus Databases. ASS.

[20] Philip Chen C.L., Zhang C.-Y. (2014). Data-Intensive Applications, Challenges, Techniques and Technologies: A Survey on Big Data. Inf. Sci..

[21] Schoder D. (2018). Introduction to the Internet of Things. Internet of Things A to Z.

[22] Krishna P.V. (2014). Challenges, Opportunities, and Dimensions of Cyber-Physical Systems.

[23] Hentout A., Aouache M., Maoudj A., Akli I. (2019). Human–Robot Interaction in Industrial Collaborative Robotics: A Literature Review of the Decade 2008–2017. Adv. Robot..

[24] Wong K.K. (2023). Cybernetical Intelligence: Engineering Cybernetics with Machine Intelligence.

[25] Orbann C., Sattenspiel L., Miller E., Dimka J. (2017). Defining Epidemics in Computer Simulation Models: How Do Definitions Influence Conclusions?. Epidemics.

[26] Zorzenon R., Lizarelli F.L., Moura D.B.A.D.A. (2022). What Is the Potential Impact of Industry 4.0 on Health and Safety at Work?. Saf. Sci..

[27] Damilos S., Saliakas S., Karasavvas D., Koumoulos E.P. (2024). An Overview of Tools and Challenges for Safety Evaluation and Exposure Assessment in Industry 4.0. Appl. Sci..

[28] Ağseren S., Şimşek S. (2024). Touch Sensors Used in Industry 4.0 to Machines in the Manufacturing Industry on Occupational Health and Safety. Sens. Rev..

[29] O’Sullivan P., Menolotto M., Visentin A., O’Flynn B., Komaris D.-S. (2024). AI-Based Task Classification With Pressure Insoles for Occupational Safety. IEEE Access.

[30] Balasubramanian S., Shukla V., Islam N., Manghat S. (2024). Construction Industry 4.0 and Sustainability: An Enabling Framework. IEEE Trans. Eng. Manag..

[31] Asad U., Rasheed S., Lughmani W.A., Kazim T., Khalid A., Pannek J. (2023). Biomechanical Modeling of Human–Robot Accident Scenarios: A Computational Assessment for Heavy-Payload-Capacity Robots. Appl. Sci..

[32] Tan C.-H., Koo A.-C., Rahmat H., Siew W.-F., Weng-Onn Cheang A., Amir Sharji E. (2023). Workplace Wellness, Mental Health Literacy, and Usage Intention of E-Mental Health amongst Digital Workers during the COVID-19 Pandemic. Int. J. Ment. Health Promot..

[33] Macháček J., Schlossarek M., Lindagato P. (2022). The Livelihood of Artisanal and Small-Scale Miners and Awareness of the Use of 3T Minerals in Rwanda—A Case Study in the Rutsiro District: A Qualitative Assessment. Int. J. Environ. Res. Public Health.

[34] Lemos J., Gaspar P.D., Lima T.M. (2022). Environmental Risk Assessment and Management in Industry 4.0: A Review of Technologies and Trends. Machines.

[35] Niehaus S., Hartwig M., Rosen P.H., Wischniewski S. (2022). An Occupational Safety and Health Perspective on Human in Control and AI. Front. Artif. Intell..

[36] Rey-Merchán M.D.C., López-Arquillos A. (2022). Occupational Risk of Technostress Related to the Use of ICT among Teachers in Spain. Sustainability.

[37] Ávila-Gutiérrez M.J., Suarez-Fernandez de Miranda S., Aguayo-González F. (2022). Occupational Safety and Health 5.0—A Model for Multilevel Strategic Deployment Aligned with the Sustainable Development Goals of Agenda 2030. Sustainability.

[38] De Felice F., Longo F., Padovano A., Falcone D., Baffo I. (2022). Proposal of a Multidimensional Risk Assessment Methodolgy to Assess Ageing Workforce in a Manufacturing Industry: A Pilot Case Study. Saf. Sci..

[39] Bavaresco R., Arruda H., Rocha E., Barbosa J., Li G.-P. (2021). Internet of Things and Occupational Well-Being in Industry 4.0: A Systematic Mapping Study and Taxonomy. Comput. Ind. Eng..

[40] Douibi K., Le Bars S., Lemontey A., Nag L., Balp R., Breda G. (2021). Toward EEG-Based BCI Applications for Industry 4.0: Challenges and Possible Applications. Front. Hum. Neurosci..

[41] Svertoka E., Saafi S., Rusu-Casandra A., Burget R., Marghescu I., Hosek J., Ometov A. (2021). Wearables for Industrial Work Safety: A Survey. Sensors.

[42] Berti N., Finco S., Battini D. (2021). A New Methodological Framework to Schedule Job Assignments by Considering Human Factors and Workers’ Individual Needs. Proc. Summer Sch. Fr. Turco.

[43] Lopez M.A., Terrón S., Lombardo J.M., González-Crespo R. (2021). Towards a Solution to Create, Test and Publish Mixed Reality Experiences for Occupational Safety and Health Learning: Training-MR. Int. J. Interact. Multimed. Artif. Intell..

[44] Zheyuan C., Rahman M.A., Tao H., Liu Y., Pengxuan D., Yaseen Z.M. (2021). Need for Developing a Security Robot-Based Risk Management for Emerging Practices in the Workplace Using the Advanced Human-Robot Collaboration Model. Work.

[45] Tasdelen A., Ozpinar A. OHS 4.0 Approach and Use Case of Indoor Positioning Systems. In Proceedings of the 2020 4th International Symposium on Multidisciplinary Studies and Innovative Technologies (ISMSIT).

[46] Robelski S., Sommer S. (2020). ICT-Enabled Mobile Work: Challenges and Opportunities for Occupational Health and Safety Systems. Int. J. Environ. Res. Public Health.

[47] Turisova R., Sinay J., Pacaiova H., Kotianova Z., Glatz J. (2020). Application of the EFQM Model to Assess the Readiness and Sustainability of the Implementation of I4.0 in Slovakian Companies. Sustainability.

[48] Géczy A., Mátyás T.D., Kámán J. (2020). Application of Grid-Eye IR Sensor for Enhanced HMI and OSH Purposes in Industry 4.0 Reflow Soldering Environment. Proceedings of the 2020 43rd International Spring Seminar on Electronics Technology (ISSE).

[49] Martinetti A., Dongen L.A.M. (2018). van Evolution of Safety in Industry 4.0: Future Opportunities Adopting Resilience and Antifragility Engineering and Virtual and Augmented Reality. Geoing. Ambient. E Mineraria.

[50] Podgórski D., Majchrzycka K., Dąbrowska A., Gralewicz G., Okrasa M. (2017). Towards a Conceptual Framework of OSH Risk Management in Smart Working Environments Based on Smart PPE, Ambient Intelligence and the Internet of Things Technologies. Int. J. Occup. Saf. Ergon..

[51] Wallace P. (2004). The Internet in the Workplace: How New Technology Is Transforming Work.

[52] Burke R.J., Cooper C.L. (2006). The New World of Work and Organizations: Implications for Human Resource Management. Hum. Resour. Manag. Rev..

[53] Cartwright S., Holmes N. (2006). The Meaning of Work: The Challenge of Regaining Employee Engagement and Reducing Cynicism. Hum. Resour. Manag. Rev..

[54] Dolobáč M. (2022). Legal Protection of Mental Health of Employees in Slovakia. Stud. Iurid. Toruniensia.

[55] Gualtieri L., Rauch E., Vidoni R. (2022). Development and Validation of Guidelines for Safety in Human-Robot Collaborative Assembly Systems. Comput. Ind. Eng..

[56] Radović-Marković M. (2021). The Transformation of Work in a Global Knowledge Economy. Entrepreneurship and Work in the Gig Economy.

[57] Lee J. (2016). The Impact of ICT on Work.

[58] Dolphin T. (2015). Technology, Globalisation and the Future of Work in Europe: Essays on Employment in a Digitised Economy.

[59] Fuglseth A.M., Sørebø Ø. (2014). The Effects of Technostress within the Context of Employee Use of ICT. Comput. Hum. Behav..

[60] Rubery J., Grimshaw D. (2001). ICTs and Employment: The Problem of Job Quality. Int’l Lab. Rev..

[61] Reiman A., Takala E.-P., Parviainen E., Kaivo-oja J. (2024). Human Work in Strategic Technology Transitions in Manufacturing. Int. J. Hum. Factors Ergon..

[62] Bugvi S.A., Mughal K.H., Jamil M.F., Kazim A.H., Shabbir A., Shahid M.U., Dar M.U., Asif M.M., Imran H. (2024). Evaluating the Work Design Readiness for Industry 4.0 Based on Personal Characteristics of Production Workers: Work Design Readiness for Industry 4.0. Int. J. Ind. Eng. Theory Appl. Pract..

[63] Balsmeier B., Woerter M. (2019). Is This Time Different? How Digitalization Influences Job Creation and Destruction. Res. Policy.

[64] El-Kady A.H., Halim S., El-Halwagi M.M., Khan F. (2023). Analysis of Safety and Security Challenges and Opportunities Related to Cyber-Physical Systems. Process Saf. Environ. Prot..

[65] Pärli K. (2022). Impacts of Digitalisation on Employment Relationships and the Need for More Democracy at Work. Ind. Law J..

[66] Costantino F., Falegnami A., Fedele L., Bernabei M., Stabile S., Bentivenga R. (2021). New and Emerging Hazards for Health and Safety within Digitalized Manufacturing Systems. Sustainability.

[67] Cette G., Nevoux S., Py L. (2022). The Impact of ICTs and Digitalization on Productivity and Labor Share: Evidence from French Firms. Econ. Innov. New Technol..

[68] Krutova O., Koistinen P., Turja T., Melin H., Särkikoski T. (2021). Two Sides, but Not of the Same Coin: Digitalization, Productivity and Unemployment. Int. J. Product. Perform. Manag..

[69] Gomes M.G., da Silva V.H.C., Pinto L.F.R., Centoamore P., Digiesi S., Facchini F., Neto G.C.d.O. (2020). Economic, Environmental and Social Gains of the Implementation of Artificial Intelligence at Dam Operations toward Industry 4.0 Principles. Sustainability.

[70] Liu Z., Xie K., Li L., Chen Y. (2020). A Paradigm of Safety Management in Industry 4.0. Syst. Res. Behav. Sci..

[71] Bordel B., Alcarria R., Robles T., González D., Rocha Á., Ferrás C., Paredes M. (2019). An Industry 4.0 Solution for the Detection of Dangerous Situations in Civil Work Scenarios. Information Technology and Systems.

[72] Marková P., Prajová V., Homokyová M., Horvathova M. Human Factor in Industry 4.0 in Point of View Ergonomics in Slovak Republic. Proceedings of the 30th DAAAM International Symposium.

[73] Polak-Sopinska A., Wisniewski Z., Walaszczyk A., Maczewska A., Sopinski P., Karwowski W., Trzcielinski S., Mrugalska B. (2020). Impact of Industry 4.0 on Occupational Health and Safety. Advances in Manufacturing, Production Management and Process Control.

[74] Barata J., Da Cunha P.R., Abramowicz W., Corchuelo R. (2019). Safety Is the New Black: The Increasing Role of Wearables in Occupational Health and Safety in Construction. Business Information Systems.

[75] Brocal F., González C., Komljenovic D., Katina P.F., Sebastián M.A. (2019). Emerging Risk Management in Industry 4.0: An Approach to Improve Organizational and Human Performance in the Complex Systems. Complexity.

[76] Fargnoli M., Lombardi M. (2020). Building Information Modelling (BIM) to Enhance Occupational Safety in Construction Activities: Research Trends Emerging from One Decade of Studies. Buildings.

[77] Sardinha L., Baleiras J., Sousa S., Lima T., Gaspar P. (2024). Decision Support System (DSS) for Improving Production Ergonomics in the Construction Sector. Processes.

[78] Erol M. (2019). Occupational health and work safety systems in compliance with Industry 4.0: Research directions. IJEBEG.

[79] Kadir B.A., Broberg O. (2020). Human Well-Being and System Performance in the Transition to Industry 4.0. Int. J. Ind. Ergon..

[80] Adem A., Çakit E., Dağdeviren M. (2020). Occupational Health and Safety Risk Assessment in the Domain of Industry 4.0. SN Appl. Sci..

[81] Adriaensen A., Decré W., Pintelon L. (2019). Can Complexity-Thinking Methods Contribute to Improving Occupational Safety in Industry 4.0? A Review of Safety Analysis Methods and Their Concepts. Safety.

[82] Cardillo E., Caddemi A. (2019). Feasibility Study to Preserve the Health of an Industry 4.0 Worker: A Radar System for Monitoring the Sitting-Time. Proceedings of the 2019 II Workshop on Metrology for Industry 4.0 and IoT (MetroInd4. 0&IoT).

[83] Lodovici M.S., Ferrari E., Paladino E., Pesce F., Frecassetti P., Aram E. (2021). The Impact of Teleworking and Digital Work on Workers and Society.

[84] Messenger J., Vargas O.L., Gschwind L., Boehmer S., Vermeylen G., Wilkens M. (2017). Working Anytime, Anywhere: The Effects on the World of Work.

[85] Berg-Beckhoff G., Nielsen G., Ladekjær Larsen E. (2017). Use of Information Communication Technology and Stress, Burnout, and Mental Health in Older, Middle-Aged, and Younger Workers–Results from a Systematic Review. Int. J. Occup. Environ. Health.

[86] Cockburn W. (2021). OSH in the Future: Where Next?. Eur. J. Workplace Innov..

[87] Ayyagari R., Grover V., Purvis R. (2011). Technostress: Technological Antecedents and Implications. MIS Q..

[88] Giusino D., De Angelis M., Mazzetti G., Christensen M., Innstrand S.T., Faiulo I.R., Chiesa R. (2022). “We All Held Our Own”: Job Demands and Resources at Individual, Leader, Group, and Organizational Levels During COVID-19 Outbreak in Health Care. A Multi-Source Qualitative Study. Workplace Health Saf..

[89] Ragu-Nathan T.S., Tarafdar M., Ragu-Nathan B.S., Tu Q. (2008). The Consequences of Technostress for End Users in Organizations: Conceptual Development and Empirical Validation. Inf. Syst. Res..

[90] Stenfors C.U.D., Hanson L.M., Oxenstierna G., Theorell T., Nilsson L.-G. (2013). Psychosocial Working Conditions and Cognitive Complaints among Swedish Employees. PLoS ONE.

[91] Stadin M., Nordin M., Broström A., Magnusson Hanson L.L., Westerlund H., Fransson E.I. (2019). Repeated Exposure to High ICT Demands at Work, and Development of Suboptimal Self-Rated Health: Findings from a 4-Year Follow-up of the SLOSH Study. Int. Arch. Occup. Environ. Health.

[92] Brod C. (1982). Managing Technostress: Optimizing the Use of Computer Technology. Pers. J..

[93] Bakker A.B., Demerouti E. (2007). The Job Demands-Resources Model: State of the Art. J. Manag. Psychol..

[94] Day A., Paquet S., Scott N., Hambley L. (2012). Perceived Information and Communication Technology (ICT) Demands on Employee Outcomes: The Moderating Effect of Organizational ICT Support. J. Occup. Health Psychol..

[95] Lazarus R.S. (1966). Psychological Stress and the Coping Process.

[96] Tarafdar M., Pullins E.B., Ragu-Nathan T.S. (2015). Technostress: Negative Effect on Performance and Possible Mitigations. Inf. Syst. J..

[97] EU-OSHA Foresight on New and Emerging Occupational Safety and Health Risks Associated with Digitalisation by 2025|Safety and Health at Work EU-OSHA. https://osha.europa.eu/en/publications/foresight-new-and-emerging-occupational-safety-and-health-risks-associated.

[98] Green F. (2004). Work Intensification, Discretion, and the Decline in Well-Being at Work. East. Econ. J..

[99] Meyer S.-C., Tisch A., Hünefeld L. (2019). Arbeitsintensivierung und Handlungsspielraum in digitalisierten Arbeitswelten–Herausforderung für das Wohlbefinden von Beschäftigten?. IndBez.

[100] Chesley N. (2014). Information and Communication Technology Use, Work Intensification and Employee Strain and Distress. Work Employ. Soc..

[101] Witschel D., Döhla A., Kaiser M., Voigt K.-I., Pfletschinger T. (2019). Riding on the Wave of Digitization: Insights How and under What Settings Dynamic Capabilities Facilitate Digital-Driven Business Model Change. J. Bus Econ..

[102] Frey C.B., Osborne M.A. (2017). The Future of Employment: How Susceptible Are Jobs to Computerisation?. Technol. Forecast. Soc. Change.

[103] Autor D.H. (2015). Why Are There Still So Many Jobs? The History and Future of Workplace Automation. J. Econ. Perspect..

[104] Davis F.D. (1989). Perceived Usefulness, Perceived Ease of Use, and User Acceptance of Information Technology. MIS Q..

[105] Trist E.L., Bamforth K.W. (1951). Some Social and Psychological Consequences of the Longwall Method of Coal-Getting: An Examination of the Psychological Situation and Defences of a Work Group in Relation to the Social Structure and Technological Content of the Work System. Hum. Relat..

[106] Acemoglu D. (2011). Skills, Tasks and Technologies: Implications for Employment and Earnings.

[107] European Factories of the Future Research Association (EFFRA), Directorate-General for Research and Innovation, European Commission (2013). Factories of the Future: Multi-Annual Roadmap for the Contractual PPP under Horizon 2020.

[108] Hopkins J. (2024). Managing the Right to Disconnect—A Scoping Review. Sustainability.

[109] Todolí-Signes A. (2019). Algorithms, Artificial Intelligence and Automated Decisions Concerning Workers and the Risks of Discrimination: The Necessary Collective Governance of Data Protection. Transf. Eur. Rev. Labour Res..

[110] Bhat Z.H., Yousuf U., Saba N. (2023). Revolutionizing Work-Life Balance: Unleashing the Power of Telecommuting on Work Engagement and Exhaustion Levels. Cogent Bus. Manag..

